# SHINGLES VACCINE UPTAKE AMONG OLDER ADULTS: IDENTIFYING EARLY, LATE, AND NON-ADOPTERS

**DOI:** 10.1093/geroni/igz038.2980

**Published:** 2019-11-08

**Authors:** Hyewon Kang, Eileen Crimmins, Jennifer A Ailshire

**Affiliations:** 1 University of Southern California, Los Angeles, California, United States; 2 Davis School of Gerontology, University of Southern California, Los Angeles, California, United States

## Abstract

Although a shingles vaccine (Zostavax) has been available since 2006, vaccination uptake has been slow. As a newly approved shingles vaccine (Shingrix) became available in 2018, understanding factors affecting acceptance and timing of the original vaccine would be useful in establishing effective strategies for greater immunization. Using the Health and Retirement study, we examined individual-level and area-level characteristics of early and late vaccine adopters, and those who were not vaccinated between 2006 and 2016. Early adopters were those who got vaccinated during the four year window after the approval of the vaccine; late vaccine adopters were those who got vaccinated from 2010 to 2016. Early adopters (13.5%) and late adopters (18.5%) comprised 32% of the sample, leaving two-thirds unvaccinated. Regression results suggest that those who received the vaccine were more likely to be socioeconomically advantaged, covered by insurance, socially active, healthy, to use other preventive vaccines, and to live in a region with more vaccinated people. Early adopters were more likely to be highly educated, affluent, and more conscientious compared to late adopters. Utilization of influenza vaccine and living in the region with the highest level of vaccination were found to be significant factors predicting early vaccine uptake. Our findings highlight the importance of outreach efforts and health care access in increasing vaccination rates.

